# Impact of Diet Supplementation and Age at Slaughter on Carcass Characteristics of Creole Goats

**DOI:** 10.3389/fvets.2021.671948

**Published:** 2021-05-25

**Authors:** Jean-Christophe Bambou, Steve Cériac, Léticia Liméa, Rémy Arquet, Bruno Bocage, Gisèle Alexandre

**Affiliations:** ^1^INRAE Unité de Recherches Zootechniques, Centre INRAE Antilles-Guyane, Paris, France; ^2^INRAE Plateforme Tropicale d'Expérimentation sur l'Animal, Centre INRAE Antilles-Guyane, Paris, France

**Keywords:** goats, meat, feeding system, carcass, supplementation

## Abstract

The objective of this study was to evaluate the effects of diet and age on finishing performances and carcass characteristics of male Creole goats. A total of 91 weaned male Creole kids [84 days old ± 7 days, 9.2 kg live weight (LW) ± 0.5 kg] were randomly allocated in a 2 × 3 experimental design. The animals were fed individually with two diets: C0: a 28-day-old *Digitaria decubens* grass alone, or C50: the same grass plus a commercial concentrate (50% of the total diet) and then slaughtered at 7, 11, or 15 months of age. Significant feeding regimen and age at slaughter effects were observed on the goat carcass characteristics. The addition of concentrate improved the average daily gain (ADG), the dressing percentage, and the conformation score (1–5 scale) from 46 to 88 g/day, 52.8 to 62.4%, and 2.2 to 4.9, respectively. Moreover, carcasses of the C0 group appeared lean with less developed fat than the C50 group and lighter than visceral fat. The meat color was significantly more affected by diet than age. Our data suggested that the production of heavy carcasses with low proportions of fat in the meat is possible in this local breed. The valorization of such a forage feeding system until 11 months of age or with the addition of concentrate from 7 to 11 months of age should be evaluated economically.

## Introduction

The role of native breeds in the improvement of the productivity of sustainable animal production in developing countries is of particular interest (1, 2). Moreover, it is essential to secure the genetic diversity of these native breeds, notably through breeding programs targeting adaptive traits to face the challenges of global changes (3). In the Caribbean region, most goat farming is based on the native Creole breed, a robust genotype mainly reared for meat and described for its good adaptive and reproductive traits (4). The Creole goat breed of Guadeloupe is characterized by an average of 1.8 kg live weight (LW) at birth, and for bucks, a mature size of 45–60 kg LW. Depending on the system, the traditional processing weight of kids is between 18 and 20 kg LW which can be reached between 6 and 18 months of age. The introduction of exotic breeds, such as Boer goats, prized for their large size, their heavier carcass, and their greater growth rate threaten Creole goat rearing despite their better adaptation to the local environment (5). Thus, according to breeders demand, one way to promote and develop the Creole breed is to maintain adequate carcass adiposity, quality attributes, and their great adaptability to the environment while improving growth performances and yield at slaughter. The two main tools to reach this objective are the management of the genetics and nutrition of the flocks (6, 7). A nutritional strategy could be implemented in the short term to increase the growth performance either by higher feeding level or by increasing the length of the finishing period with forage only. Several studies have shown that goats fed concentrate-based diets have higher growth rates, dressing percentage, and carcass quality than goats that are grass-fed (7, 8). Therefore, feedlot production largely relies on diets rich in highly digestible energy and protein, dietary energy being considered as the baseline requirement as it affects the utilization of other nutrients (9, 10). However, the use high concentrate diets to improve traditional slaughter weight may result in fatter carcasses as reported for sheep (11, 12). In contrast, forage feeding would extend the growth period and potentially affect carcass traits. Therefore, since studies investigating meat potential ability and the effect of intensive feedlot systems and slaughter conditions on this tropical breed are scarce, the effects of diet and age at slaughter on growth performance and carcass traits should be assessed. Therefore, the main objective of this study was to assess the effects of diet and age at slaughter on the finishing performance and carcass characteristics of male Creole goats, and the fatty acid composition of main adipose and muscle tissues.

## Materials and Methods

This study was carried out in the INRAE PTEA (Plateforme Tropicale d'Expérimentation sur l'Animal) experimental farm in Guadeloupe, a humid tropical island in the Caribbean (16.1°N, 61.6°W). The experimental farm is located in the driest region of the island where annual rainfall averages 1,280 mm, with a dry season lasting from January to May with <70 mm per month. Maximum air temperatures vary from 27°C (January) to 32°C (August) with a minimum from 21 to 25°C, respectively. The relative humidity is usually above 70% and the day length ranges from 11 to 13 h. In accordance with the current law on animal experimentation and ethics, this experiment was evaluated by an ethical committee and approved by the French Ministry of Agriculture after evaluation by the Animal Care and Use Committee of French West Indies and Guyana (authorization number: 69-2015-1).

### Animal Management and Experimental Groups

A total of 91 Creole male kids were used in this experiment in a completely randomized design with a complete 2 × 3 experimental design (feeding regimen × age at slaughter). The kids were reared at pasture from birth until weaning with their mother in a rotational grazing system. The pasture was divided into five plots grazed by 50–52 suckling goats with their progeny until weaning. Each plot was grazed for 1 week and rested for 4 weeks.

After weaning, at 84 days old ± 7 days and 9.2 kg live weight (LW) ±0.5 kg, kids were allocated by birth weight, weaning weight, pre-weaning average daily gain (ADG), and the LW of the doe, and randomly assigned to experimental groups. Animals were reared indoors in individual pens on a slatted floor. In group C0 (no concentrate, *n* = 45), kids received no commercial concentrate and in group C50, they received a commercial pellet in addition to their basal forage diet (50% of the diet, *n* = 46).

Ages of the animals at slaughter were in 4-month increments from weaning at 3 months old according to a 4-month interval of either kidding or weaning of the experimental flock: ages at slaughter were 7 months (i.e., age at weaning plus 4 months; *n* = 15 C0 and *n* = 15 C50), 11 months (*n* = 16 C0 and *n* = 17 C50), and 15 months (*n* = 14 C0 and 14 C50).

### Diets and Measures

The basal diet was *Digitaria decubens* allocated *ad libitum* and cut daily to achieve a 28-day regrowth: 0.74 UFL (feed unit for lactation) and 79 g PDIN (ruminally degradable nitrogen) per kg DM (dry matter). The pasture was divided into 29 plots and the harvest was planned so that the whole experimental period would fall within a *D. decubens* regrowth age of 28 days. One plot was harvested per day. The pasture was irrigated and fertilized with 500 kg/ha/year of commercial fertilizer (30% N, 12% P_2_O_5_, and 18% K_2_O). The commercial concentrate (1.15 UFL and 150 g PDIN per kg DM) was composed of maize (68%), soybean cake (15%), wheat bran (11%), vitamin and mineral supplement (5%), and urea (1%).

The animals had free access to fresh tap water and mineral blocks composed of sodium (32.5%), calcium (2.5%), and magnesium (2.5%). The animals were allowed to adapt to their diets and pens for 14 days before starting the measurements. The animals received the commercial concentrate first at 0600 h and then the forage was distributed in two meals: at 0800 and 1400 h. The amount of forage offered was 1.15 times higher than the voluntary intake measured the days before. Voluntary feed intake was measured from Monday to Friday during the experiment. Offered and refused forage were recorded weekly for each animal to measure voluntary dry matter intake. Samples of offered forage (two sub-samples of 200 g) and refusals (10% for each animal) were collected daily. One of the sub-samples was kept for daily dry matter determination. All the samples of the feed provided during the 2 weeks were mixed together and a new sub-sample (200 g) was used for chemical analyses. The same operation was carried out for feed refusals.

### Slaughtering Procedure

Animals were weighed the day before slaughter, and the next day while fasting just prior to slaughter. The animals were humanely slaughtered with a captive bolt pistol which hit them on the head producing immediate unconsciousness following by exsanguination. Thereafter, the digestive tract was removed, weighed in full, separated by compartment, emptied, and then re-weighed. The omental and mesenteric fat were removed and weighed. Weights of non-carcass components were recorded. Dressed carcasses were weighed within 1 h of slaughter (hot carcass weight: HC), stored at 4°C for 24 h, and then weighed (cold carcass weight: CC). Cold carcasses were rated by three experts (from 1 to 5) according to conformation and internal and external fat based on a lightweight lamb grid (13). The perirenal fat (PR) was removed and weighed. The carcass was then cut in half lengthwise and the left side was cut according to Colomer-Rocher et al. (14) into five joints (shoulder, neck, ribs, flank, and long leg). Every joint was weighed and the left shoulder was dissected into fat (subcutaneous plus intermuscular deposits, IM deposits), muscles, and bones.

The right shoulder was removed and frozen at −80°C until the end of the experiment, then cut while frozen with a MAGURIT machine (Unitcut 545 SC model), ground using a 3-mm grid (BIRO AFMG 48/52, BIRO France), and homogenized. Aliquots were freeze-dried and were finely ground in a ball grinder (Dangoumill 300, Prolabo, Paris, France) using liquid nitrogen for chemical analysis.

Samples of muscle (supraspinatus, SE) and intermuscular (IM) adipose tissues of the shoulder and samples of perirenal (PR) fat were frozen at −80°C until analysis.

### Chemical Analyses

DM concentrations of both allocated and refused forage were determined by drying samples to a constant weight at 65°C in a ventilated drying oven. The samples were then ground (0.75 mm) prior to analysis. The forage and carcass samples were analyzed according to AFNOR procedures (15): dry matter (DM, AFNOR NF V18-109), ash (AFNOR NF V18-101), crude protein (CP, N x 6.25, AFNOR NF V18-120), and ether extract (EE, AFNOR NF V18-117). Neutral detergent fiber (NDF), acid detergent fiber (ADF), and acid detergent lignin (ADL) were determined according to Van Soest et al. (16).

The DM of PR, IM, and SE were determined after freeze-drying for 48 h. The lipid extract concentrations of PR, IM, and SE were determined by ether-petroleum extraction. The fatty acids (FA) extraction was carried out according to the method described by Rule (17), but using tricosanoic acid as an internal standard in the extraction solvent mixture. Lipid extraction and methylation were described by Bas et al. (18). Samples were then injected with auto sampler CP-8410 into a Varian CP-3900 gas liquid chromatograph (GLC, Varian, Les Ulis, France) on a DB-wax-fused silica capillary column (60 m × 0.25 mm i.d. × 0.25 μm film thickness: JW, Folsom, CA). For the GLC procedure, the split/split less injector type 1177, and the flame ionization detector were held at 250°C. The oven temperature increased from 120 to 195°C at a rate of 4°C/min, and then held for 60 min at constant temperature. The injector was in splitless mode for 1.0 min and then in split mode until the end of the run with a split ratio of 30:1. The column flow rate was 1.2 mL/min of He. FA were identified by comparison to reference standards (Fatty Acid Methyl Ester, FAME, Sigma, St. Louis, MO; Interchim, Montlucon, France) and analyzed under similar conditions. Under these conditions, some positional isomers, notably trans-octadecenoic acid, could not be separated from each other (19).

Several evaluations were made on the cold carcasses. The color and ultimate pH of the rib eye area of the fourth rib on the left side was evaluated, respectively with Minolta chromameter CR-300 calibrated to a white standard, using the L^*^, a^*^, b^*^ scale, and a Bioblock Scientific IP67 pH probe (Fischer scientific, Illkirch, France) calibrated to pH 4 and 7 using buffer standards.

### Data Calculations and Statistical Analyses

Empty body weight (EBW) was calculated by subtracting the weight of the gut content from the slaughter weight. The carcass yield was the weight of cold carcass related to EBW.

The FA were summed by families according to their structure: even straight-chain saturated FA (**ESFA**) = C10:0 + C12:0 + C14:0 + C16:0 + C18:0 + C20:0 + C22:0 + C24:0; odd-numbered straight-chain FA (**odd FA**) = C13:0 + C15:0 + C17:0 + C17:1*n*−8 + C19:0 + C21:0; methyl-branched chain FA of the iso and anteiso forms, (**isoFA** and **anteisoFA**) = isoC14:0 + isoC15:0 + anteisoC15:0 + isoC16:0 + isoC17:0 + anteisoC17:0; straight-chain monounsaturated FA (**MUFA**) = C14:1 + ∑C16:1 (*n*−9 and *n*−7) +∑C18:1 (*n*−7, *n*−9, trans-10) + C17:1*n*−8 + C20:1 + C22:1 + C24:1; *n*−3 FA = C18:3*n*−3 + C20:3*n*−3 + C20:5*n*−3 + C22:5*n*−3 + C22:6*n*−3; *n*−6 FA= C18:2*n*−6 + C18:3*n*−6 + C20:2*n*−6 + C20:3*n*−6 + C20:4*n*−6 + C22:4*n*−6; and polyunsaturated FA (**PUFA**) = *n*−3 FA + *n*−6 FA.

Data were analyzed using PROC GLM (Version 9, SAS Inst., Inc., Cary, NC, 1999) with diet, age at slaughter, and interaction diet^*^age as the main effects in the model. The statistical unit was the animal. Initial LW and slaughter weight were used as co-variables for ADG and carcass traits. Co-variables were kept in the model when significant. Carcass quality scores and cut weights were studied with carcass weight used as a co-variable, which was only kept in the model when significant.

## Results

The compositions of the forage and the commercial concentrate are shown in Table 1. The concentration of C18:3 FA was higher in the forage, whereas the concentration of C16:0, C18:1*n*−9, and C18:2*n*−6 was higher in the commercial concentrate.

**Table 1:** Chemical composition of experimental diets (g/kg DM).

**Diets**	**Concentrate^**a**^**	**Tropical forage**
DM^1^	889	256
**g/kg DM**		
CP^2^	209	138
EE^3^	25	14
NDF^4^	168	702
ADF^5^	47	337
ADL^6^	17	39
C14:0	0.10	0.12
C16:0	4.55	1.53
C16:1	0.04	0.01
C18:0	0.66	0.25
C18:1c9	5.16	0.28
C18:2c9c12	3.51	0.90
C18:3c9c12c15	0.15	1.13
**Feeding value**^**b**^ **per kgDM**		
UFL^7^	1.15	0.73
PDIN^8^ (g)	151	78
PDIE^9^ (g)	150	90
ME^10^ (KJ/Kg DM)	8.3	5.3

a *Composition, per kg as fed basis: 680 g of corn grain, 150 g of soybean cake, 110 g of wheat bran, 10 g of urea, and 50 g vitamin and mineral supplement*.

b *According to INRA feeding system*.

1 *DM, Dry matter;*

2 *CP, Crude protein;*

3 *EE, Ether extract;*

4 *NDF, Neutral detergent fiber;*

5 *ADF, Acid detergent fiber;*

6 *ADL, Acid detergent lignin;*

7 *UFL, Feed unit for lactation;*

8 *PDIN, Ruminally degradable nitrogen;*

9 *PDIE, Ruminally degradable energy;*

10 *ME, Metabolizable energy*.

### Growth and Carcass Performances

The addition of 50% of concentrate to the diet significantly increased DMI, ADG, feed conversion ratio (FCR), and carcass weights, yields, and scores but not the color (*P* < 0.01, Table 2). A significant effect of age was observed for DMI and FCR but not for ADG (*P* < 0.05). The FCR was affected only by the interaction diet × age (*P* < 0.05). The ADG increased by 80% in the C50 compared with the C0 group (*P* < 0.05). The differences between the C50 and the C0 groups in carcass traits were high: addition of concentrate accounted for 75–80% increases in the weight of hot and cold carcasses, prime cuts, and muscle and 50% increases for conformation scores. Finally, the dressing percentage increased with the addition of concentrate, although to a lesser extent (10%). The non-carcass components parameters were significantly affected by the diet except for the proportion of red organs (related to EBW) and retail cuts (related to carcass weight; *P* < 0.05, Table 2). In addition, abdominal fat tissues and neck proportions increased by 30% with age at slaughter (*P* < 0.05).

**Table 2:** Growth performances and carcass traits of Creole goats according to diet and age at slaughter.

**Groups**	**C0^1^**			**C50^2^**				**Significance^4^**		
**Age at slaughter (months)**	**7**	**11**	**15**	**7**	**11**	**15**	**SEM^3^**	**D**	**A**	**D × A**
*N*	16	16	13	15	16	15				
ADG^5^ (g/d)	46.4	46.6	46.5	83.5	87.7	78.4	3.42	^**^	NS	NS
Total DM^6^ intake (g/d)	532	554	509	603	681	707	29.3	^**^	^*^	NS
Feed conversion ratio	11.5	11.9	10.9	7.2	7.8	9.0	0.18	^**^	^*^	^*^
Slaughter weight (kg)	16.0	20.5	25.5	19.2	30.5	36.5	3.6	^**^	^**^	NS
EBW^7^ (kg)	10.5	13.2	18.1	14.8	24.4	29.5	2.7	^**^	^**^	NS
Hot carcass weight (kg)	5.8	7.4	10.6	8.6	15.1	18.7	1.8	^**^	^**^	NS
Cold carcass weight (kg)	5.5	7.1	10.3	8.4	14.8	18.4	1.8	^**^	^**^	NS
Dressing percentage (%)	52.8	53.8	57.0	57.1	60.7	62.4	2.7	^**^	^**^	NS
Conformation (1–5)	2.2	2.7	3.4	3.3	4.3	4.9	0.9	^**^	^**^	NS
External fat (1–5)	1.5	1.8	2.1	3.1	3.1	2.9	0.6	^**^	^**^	NS
Internal fat (1–5)	1.7	2.3	2.8	3.1	3.5	4.8	0.7	^**^	^**^	NS
Color (1–4)	1.6	2.1	2.6	1.7	1.9	3.0	0.4	^*^	^*^	NS
**Non-carcass components**										
Omental fat (g)	73	132	201	211	568	946	171	^**^	^**^	NS
Perirenal fat (g)	48	78	112	122	326	599	144	^**^	^**^	NS
Mesenteric fat (g)	170	198	273	236	466	650	153	^**^	^**^	NS
Total fat (% EBW)	2.8	3.1	3.2	3.8	5.6	7.2	0.9	^**^	^**^	NS
Empty GI- tract (% EBW)	11.8	11.5	9.3	9.6	6.5	5.6	1.60	^**^	^**^	NS
Red organs (%)	5.4	5.0	4.3	5.2	4.2	4.0	0.90	NS	^*^	^*^
**Primal cuts**										
Shoulder (%)	19.6	19.4	19.2	20.1	19.4	18.1	0.7	NS	NS	NS
Neck (%)	11.0	12.3	13.7	11.4	14.7	15.4	1.8	NS	^**^	NS
Long leg (%)	32.1	30.8	29.9	31.4	28.8	27.6	1.5	NS	^**^	NS
Ribs + loin (%)	22.0	22.3	22.7	22.3	23.0	23.1	1.4	NS	NS	NS
Flank (%)	15.3	15.2	14.4	14.8	14.3	14.8	1.3	NS	NS	NS
**Left shoulder components**										
Muscle (%)	69.1	70.8	73.7	71.3	74.2	75.1	9.62	^*^	^*^	NS
Bone (%)	25.2	23.2	21.2	20.7	19.0	17.9	3.03	^*^	^**^	NS
Fat (%)	5.6	5.9	5.1	7.9	6.8	7.0	2.07	^**^	^*^	NS
Muscle/bone ratio	2.8	3.1	3.5	3.5	3.9	4.2	0.47	^**^	^*^	NS

1 *C0: Creole goats fed with 28-day-old Digitaria decubens grass alone; n = 15, 16, and 14 kids respectively at 7, 11, and 15 months of age*.

2 *C50: Creole goats fed with a 28-day-old Digitaria decubens grass plus a commercial concentrate (50% of the total diet); n = 15, 17, and 14 respectively at 7, 11, and 15 months of age*.

3 *SEM, Standard error of the mean*.

4* *P < 0.05;*

** *P < 0.01, D: animal group (C0 and C50), A: age at slaughter (7, 11, and 15 months old)*.

5 *ADG, Average daily gain*.

6 *DM, Dry matter*.

7 *EBW, Empty body weight*.

The intermuscular fats were ~2-fold greater in the C50 compared to C0 group (*P* < 0.05). The proportions of muscle ranged from 70 to 77% within the groups. The muscle/bone ratio was significantly affected by age at slaughter and diet (*P* < 0.05) but no interaction between these two effects was observed.

### Quality Parameters of Carcass, Fat, and Muscle Tissues

With the exception of traits related to collagen and glycogen analyses, a significant diet effect was observed on almost all parameters (*P* < 0.05, Table 3). The pH values, water losses, and “L” and b” parameters were higher in C0 compared with C50 kids (*P* < 0.05). The shoulder DM and ether extract concentrations increased in the C50 group and with age while ash declined (*P* < 0.05). Interactions were observed for the color parameter “a” measured in the longissimus dorsi and the shoulder CP concentration. A significant effect of age was observed for collagen solubility (%) which decreased with time (*P* < 0.05).

**Table 3:** Physical measurements on muscle and chemical analyses on shoulder and muscles of Creole goats according to diet and age at slaughter.

**Groups**	**C0^1^**			**C50^2^**				**Significance^4^**		
**Age at slaughter (months)**	**7**	**11**	**15**	**7**	**11**	**15**	**SEM^3^**	**D**	**A**	**D × A**
**Physical measures on longissimus dorsi**										
Ultimate pH	5.6	6.9	6.3	5.9	6.1	5.5	0.5	^*^	NS	^*^
Cooking loss, %	24.7	21.0	25.4	22.4	29.0	28.9	6.7	^*^	NS	^**^
**Color**										
L^5^-lightness	48.5	46.4	41.0	43.4	41.7	37.9	9.0	^**^	^**^	NS
a^5^-redness	16.9	17.3	17.8	16.3	16.7	17.2	2.7	NS	NS	^**^
b^5^-yellowness	6.8	5.8	5.5	5.1	5.4	6.2	1.0	^*^	NS	NS
**Chemical composition of shoulder**										
DM^6^ (%)	32.0	30.9	36.2	31.9	34.1	49.2	5.1	^**^	^**^	NS
Ash (g kg^−1^DM^7^)	19.7	17.9	15.6	14.4	16.0	12.2	2.7	^**^	^**^	NS
CP^7^ (g kg^−1^DM^7^)	60.8	62.1	62.0	61.0	59.0	59.3	3.8	NS	NS	^*^
EE^8^ (g kg^−1^DM^7^)	20.4	20.5	21.3	25.9	24.2	28.8	4.6	^**^	^*^	NS
**In longissimus dorsi muscle**										
Soluble collagen, mg/g	42.1	30.1	15.3	30.1	19.3	12.7	12.97	NS	^**^	NS
Insoluble collagen, mg/g	15.2	14.0	19.6	13.7	15.7	15.3	3.62	NS	NS	NS
Collagen solubility, %	72.9	60.1	41.2	68.4	54.0	41.6	13.52	NS	^**^	NS
**In supraspinatus muscle**										
Glycogen content, μmol/g	1.9	2.3	4.7	4.1	4.4	3.2	3.75	NS	NS	NS
Lactate content, μmol/g	60.1	64.9	75.3	67.7	68.8	63.1	13.50	NS	^*^	NS
GP^9^, μmol/g eq lactate	79.8	68.7	69.1	75.8	77.7	69.5	18.3	NS	NS	NS

1 *C0: Creole goats fed with 28-day-old Digitaria decubens grass alone; n = 15, 16, and 14 kids respectively at 7, 11, and 15 months of age*.

2 *C50: Creole goats fed with 28-day old D. decubens grass plus a commercial concentrate (50% of the total diet); n = 15, 17, and 14 kids respectively at 7, 11, and 15 months of age*.

3 *SEM, Standard error of the mean*.

4* *P < 0.05;*

** *P < 0.01. D: animal group (C0 and C50), A: age at slaughter (7, 11, and 15 months old)*.

5 L^*^, a^*^, b^*^ scale of the Minolta chromameter CR-300 calibrated to a white standard.

6 *DM, Dry matter;*

7 *CP, Crude protein;*

8 *EE, Ether extract;*

9 *GP, Glycolytic potential*.

### Fatty Acid Composition of Adipose Tissue and Muscles

Perineal and intramuscular adipose tissue DM concentrations were affected by diet (*P* < 0.01, Table 4). The DM concentrations of intermuscular adipose tissue were affected by diet (*P* < 0.01) and age at slaughter (*P* < 0.05) whereas only age at slaughter had an effect on DM concentrations of supraspinatus muscle tissue (*P* < 0.01). In addition, the DM concentrations of intermuscular adipose tissue increased more sharply with age at slaughter in the C50 group, resulting in a diet × age interaction (*P* < 0.01). The age at slaughter had a significant effect on lipid concentrations of perineal and intermuscular adipose tissues but no effect of diet was observed (*P* < 0.01 and 0.05, respectively).

**Table 4:** Major fatty acid (FA) proportions (g/100g of FA) in adipose tissues, perirenal and intermuscular fats, and muscle tissue of Creole goats according to diet and age at slaughter.

**Groups**	**C0^1^**			**C50^2^**				**Significance^4^**		
**Age at slaughter (months)**	**7**	**11**	**15**	**7**	**11**	**15**	**SEM^3^**	**D**	**A**	**D × A**
**In perirenal adipose tissue**										
DM, %	66.6	68.7	77.2	89.1	91.6	91.8	5.90	^**^	NS	NS
Lipid content, %	88.0	95.9	98.6	94.4	98.7	97.4	7.43	NS	^**^	NS
SFA^5^	53.1	55.6	55.3	53.9	54.7	49.9	2.99	^*^	NS	^*^
MUFA^6^	32.8	27.7	28.6	31.9	30.8	36.3	3.32	^**^	^**^	^**^
PUFA^7^	4.6	4.4	4.3	5.0	5.3	5.1	0.63	^**^	NS	NS
Odd chain FA^8^	3.8	4.4	4.4	2.8	2.5	2.7	0.30	^**^	NS	^**^
*Iso* and *anteiso* FA^9^	3.3	4.9	4.7	3.0	3.3	2.8	0.52	^**^	^**^	^**^
*n*−3^10^	1.1	1.0	1.0	0.6	0.6	0.6	0.18	^**^	NS	NS
*n*−6^11^	2.4	2.5	2.4	3.5	4.0	3.6	0.49	^**^	NS	NS
*n*−6/*n*−3	2.4	2.5	2.4	5.6	6.7	6.5	0.59	^**^	^**^	^**^
**In intermuscular adipose tissue**										
DM, %	48.3	56.0	57.0	49.9	70.8	72.0	18.29	^**^	^*^	^**^
Lipid content, %	65.3	73.6	75.9	68.7	83.7	87.7	19.16	NS	^*^	NS
SFA	52.6	51.1	49.2	42.8	48.8	47.8	4.96	^**^	NS	^*^
MUFA	33.7	34.8	36.9	43.0	39.0	39.8	4.95	^**^	NS	NS
PUFA	4.6	4.5	4.8	4.5	4.8	4.8	0.66	NS	NS	NS
Odd chain FA	3.8	4.0	3.8	3.9	2.7	2.7	0.64	^**^	^*^	^**^
*Iso* and *anteiso* FA	3.2	3.5	3.3	2.5	2.3	2.4	0.47	^**^	NS	NS
*n*−3	1.1	1.0	1.1	0.6	0.5	0.5	0.21	^**^	NS	NS
*n*−6	2.4	2.2	2.5	2.7	3.3	3.3	0.51	^**^	NS	^*^
*n*−6/*n*−3	2.4	2.3	2.7	5.0	6.3	6.7	0.78	^**^	^**^	^**^
**In supraspinatus muscle tissue**										
DM, %	37.6	23.0	30.6	36.3	24.3	29.8	10.51	NS	^**^	NS
Lipid content, %	12.2	10.1	10.7	11.3	11.2	11.5	3.96	NS	NS	NS
SFA	41.4	41.2	41.9	40.9	41.4	41.5	3.37	NS	NS	NS
MUFA	35.9	37.9	37.8	40.1	38.9	38.8	2.48	^**^	NS	^*^
PUFA	10.7	9.7	9.5	9.8	11.0	11.3	2.01	NS	NS	NS
Odd chain FA	3.4	3.8	4.0	3.0	2.5	2.6	0.40	^**^	NS	^**^
*Iso* and *anteiso* FA	2.5	2.7	2.9	1.9	2.0	2.2	0.35	^**^	^*^	NS
*n*−3	2.6	2.1	2.5	1.0	0.9	0.8	0.43	^**^	NS	NS
*n*−6	7.2	6.5	5.9	7.9	9.4	9.6	1.87	^**^	NS	^*^
*n*−6/*n*−3	3.2	3.1	2.3	7.8	11.2	11.6	1.62	^**^	^**^	^**^

1 *C0: Creole goats fed with 28-day-old Digitaria decubens grass alone; n = 15, 16, and 14 kids respectively at 7, 11, and 15 months of age*.

2 *C50: Creole goats fed with 28-day-old Digitaria decubens grass plus a commercial concentrate (50% of the total diet); n = 15, 17, and 14 kids respectively at 7, 11, and 15 months of age*.

3 *SEM, Standard error of the mean*.

4* *P < 0.05;*

** *P < 0.01. D: animal group (C0 and C50), A: age at slaughter (7, 11, and 15 months old)*.

5 *SFA, Saturated fatty acids (g/100g of FA);*

6 *MUFA, Monounsaturated fatty acids (g/100g of FA);*

7 *PUFA, Polyunsaturated fatty acids (g/100g of FA)*.

8 *Odd chain FA*.

9 *Iso and anteiso FA = isoC14:0 + isoC15:0 + anteisoC15:0 + isoC16:0 + isoC17:0 + anteisoC17:0*.

10 *n−3 FA = C18:3c9c12c15 +C20:3c11c14c17 +C20:5c5c8c11c14c17 +C22:5c7c10c13c16c19 +C22:6c4c7c10c13c16c19*.

11 *n−6 FA = C18:2c9c12 + C18:3c6c9c12 + C20:2c11c14 + C20:3c8c11c14 + C20:4c5c8c11c14 + C22:4c7c10c13c16*.

The FA compositions of perineal, intermuscular, and supraspinatus adipose tissues were significantly affected by diet and/or age at slaughter (*P* < 0.05, Table 4). Proportions of saturated fatty acid (SFA), odd chain FA, iso and anteiso FA, and *n*−3 FA in perineal adipose, intermuscular adipose, and supraspinatus muscle tissues were higher in the C0 group, with the exception of SFA in supraspinatus muscle tissue (*P* < 0.01). Furthermore, this effect was more pronounced with increasing slaughter age. In contrast, proportions of monounsaturated FA (MUFA) and *n*−6 and *n*−6/*n*−3 FA were higher in the C50 group in perineal and intramuscular adipose tissue (*P* < 0.05), and in supraspinatus muscle tissue (*P* < 0.01). Proportions of polyunsaturated FA were higher in perirenal adipose tissue of C50 goats (*P* < 0.01) with no effect of age at slaughter, whereas no effect was observed in intermuscular adipose tissue and supraspinatus muscle tissue. The composition of the supraspinatus muscle tissue in the major FA C16:0 and C18:0 was affected by diet and age at slaughter whereas only diet influenced C18:1*n*−9 (*P* < 0.05); nonetheless no effect of diet × age at slaughter was observed. Effects of diet, age at slaughter, and the interaction diet × age at slaughter were observed in the proportions of C20:0, C17:1*n*−8, C20:3*n*−6, C20:4*n*−6, and C20:5*n*−3 FA in supraspinatus muscle tissue (*P* < 0.05, Table 4). With the exception of C20:4*n*−6 FA, these FA were significantly higher in the C0 group. In addition, the differences were more important as the age at slaughter increased. In the present study, C18:1cis-11, C18:1trans-11, and other positional isomers of C18:1trans FA could not be quantified separately because they co-eluted on the column used (19).

## Discussion

Genetic variation which could be viewed as a consequence of the Red Queen hypothesis has led to the large diversity of locally adapted breeds worldwide (20). More largely, to face the objectives of livestock production in the context of global changes, it is essential to secure genetic diversity through conservation and breeding programs targeting adaptive traits in locally adapted breeds (21, 22). Indeed, genetics is one of the most important factors influencing production traits, but environmental factors such as diet are also important factors influencing production traits, notably meat (23). In the tropics, the most frequent feeding systems for small ruminants are based on tropical forages, considered in some studies as a limiting factor for the expression of the productive potential in some breeds because of their low to medium nutritive value (24). Thus, the improvement of the nutritional level remains necessary to increase animal performances. The present experiment was designed to measure the effect of supplementation in a forage feeding system on Creole buck performances. We evaluated the influence of age at slaughter since forage finishing result in lower growth rate and consequently longer fattening periods.

As expected, the inclusion of 50% concentrate in the diet was responsible for ~80% of the increase of the ADG and slaughter performances (i.e., 75–80% of increases for the weights of the carcasses, prime cuts, and muscles and 50% for conformation score), but the range of variations observed were relatively large compared to data reported for other tropical breeds (5, 25–27). It is largely admitted that for tissue partitioning and consequently their effects on quality, the maximum growth rate is reached firstly by bone, secondly by muscle, and lastly by fatty tissue (28). In our experimental conditions, after 1 year of intensive feeding our results suggested that in Creole kids, muscle mass reached upper levels but intramuscular fat deposits were not at maximum. The DMI relative to the metabolic weight (70.5–72.0 g kg LW-0.75) of the Creole kids was in accordance with the values reported for other tropical breeds fed under semi-intensive conditions (71 and 80 g kg LW-0.75) (26, 29, 30). In accordance with these results and the curvilinear trend observed with a threshold value for the supplemented kids, we concluded that the fattening growth potential of Creole goats would be 85 g d^−1^. It has been shown in other tropical goats that higher values (92–148 g d^−1^) could be reached with high-energy diets (29–32). However, the relative growth rate (i.e., the ratio of fattening ADG to birth weight) reached 4.5% for this Creole breed and were very similar to those of Omani and crossbred Boer goats (4.1 and 4.3%, respectively) (33, 34).

The carcass yields reported here were in the upper range of the values reported for other breeds reared in similar conditions (52 and 42% when related to LW and 60 and 54% when related to EBW, for the supplemented and non-supplemented group, respectively) (25, 29, 32, 35, 36). In keeping with previous studies, supplementation mostly impacted the non-carcass components with increased visceral and organ weights (data not shown) whereas their proportion to EBW was inversed (31, 37). Since the variables are related to EBW, this measurement was lower for forage-fed kids with a higher mass of gut fill. Moreover, in accordance with previous studies, the stomach of forage-fed kids would be heavier because of the high digestive activities related to the digestion of a high fiber diet (29, 37).

In contrast with other studies, one of the most interesting results in our study was the lower range of variations observed (5, 29), except for the carcass of supplemented kids due to the additional effect of age in fat deposition. Generally, in sheep the use of forage-feeding systems is recommended to avoid the detrimental effect of an intensive feeding system on carcass scoring which generates fatter carcasses (12). In the present study with Creole goats, the carcasses had highly acceptable fat cover scores and internal fat weights whatever the feed level. In goats, fat deposits are more important in the abdominal cavity (38). This apparent ability of Creole kids to deposit less external fat is thus of great interest for the valorization of the carcasses.

On the other hand, the wide improvement of the carcass conformation observed for kids fed with the energy-rich diet was in keeping with the demand of the local sector for heavy and well-conformed goat carcasses (13). The lower fat content (5–7%) of the Creole goat carcass could explain the high muscle percentage recorded compared to the 10–13% observed by other studies (33, 39). In the shoulder, the total lipid contents and the DM increased significantly with the feeding level and age while ash decreased. These variations are in line with those of bone and fat proportions, which have already been reported (25, 31, 40, 41). In accordance with previous studies in sheep and goats, the muscle/bone ratio increased with supplementation (12, 42, 43).

As reported for sheep and goats, here the pH values for LD muscle were relatively high in kids fed on the forage diet (36, 44–46). In contrast with previous studies, the cooking loss varied according to the feeding levels of kids (47, 48). However, it has been shown that variations could be due to genotype and could explain the differences between studies (49, 50). In the present study, the lower cooking loss was associated with lower pH and greater fat proportions observed in supplemented kids.

Concerning health aspects of SFA, goat meat composed of IM and muscle tissues was not different between supplemented and non-supplemented kids. There is no consensus on the impact of concentrate diets in goats on SFA proportions in muscles. Some studies reported that the SFA proportions were not affected by concentrate levels in the intramuscular lipids of goats, whereas others showed significant increases or decreases in the muscle of goats fed an increasing percentage of concentrate or grain fed-goats compared to range goats (5, 44, 51–53). In Creole goats, higher MUFA and PUFA, considered as desirable FA, were found in SE muscle. However, as reported by Sikora et al. (54) MUFA in muscles of the oldest animals were higher. In agreement with previous studies in goats, in all tissues the total odd FA plus iso and anteiso FA proportions ranged from 2.3 to 4.4% of the total FA and a decrease in odd and methyl branched-chain FA proportions was associated with an increase of the concentrate level (44, 52, 55). The total *n*−3 FA in muscle and PR were in accordance with the results observed in goats fed with argan pulp but lower than those observed in browsing goats without supplementation (5, 55). The discrepancies in *n*−3 FA proportion between these studies could be due to the differences between the C18:3*n*−3 content of the diets used in the experiments as well as in the ruminal physico-chemical conditions that affect the biohydrogenation of PUFA in the rumen (56).

In conclusion, in this study it has been shown that a local Creole breed known for its lean carcasses has the potential to produce heavier carcasses while maintaining low proportions of fat in the meat. The feeding mode and the age at slaughter greatly influenced the carcass traits. The valorization of a forage feeding system until 11 months of age or with the addition of concentrate from 7 to 11 months of age should be evaluated economically.

## Data Availability Statement

The raw data supporting the conclusions of this article will be made available by the authors, without undue reservation.

## Ethics Statement

The animal study was reviewed and approved by Comité d'Ethique en Matière d'Expérimentation Animale des Antilles et de la Guyane, C2EA-69.

## Author Contributions

All authors listed have made a substantial, direct and intellectual contribution to the work, and approved it for publication.

## Conflict of Interest

The authors declare that the research was conducted in the absence of any commercial or financial relationships that could be construed as a potential conflict of interest.
